# Predictive value of ALBI score and age for developing resistance to trastuzumab in HER-2-positive breast cancer patients: prediction based on a real-world case series from a single center in China

**DOI:** 10.3389/fphar.2026.1679399

**Published:** 2026-03-06

**Authors:** Jiaqi Long, Yujing Qin, Kezhen Qi, Jingwei Li

**Affiliations:** 1 The First Clinical College of Shandong University of Traditional Chinese Medicine, Jinan, Shandong, China; 2 Department of Thyroid and Breast Surgery, Affiliated Hospital of Shandong University of Traditional Chinese Medicine, Jinan, Shandong, China

**Keywords:** ALBI score, drug resistance, HER-2 positive breast cancer, single center analysis, trastuzumab

## Abstract

**Objective:**

To assess the clinical value of the ALBI score and age in predicting trastuzumab resistance in patients with HER-2 positive breast cancer (BC).

**Methods:**

A retrospective cohort study was conducted on patients with HER-2 positive BC treated with trastuzumab at the Department of Thyroid and Breast Surgery of Affiliated Hospital of Shandong University of Traditional Chinese Medicine from December 2017 to December 2023. Patients were divided into a resistance group and a non-resistance group based on the development of resistance after trastuzumab treatment. Multivariate logistic regression models were used to identify independent predictors of trastuzumab resistance, and ROC curves analysis was conducted to evaluate the predictive value of ALBI score and patients age.

**Results:**

This study included 95 female patients with HER-2 positive BC treated with trastuzumab, aged 31–71 years (mean age 53.15 ± 10.063 years). Based on the development of resistance after trastuzumab treatment, patients were divided into a resistant group (11 cases, 11.6%) and a non-resistant group (84 cases, 88.4%). Compared with the non-resistant group, the resistant group showed significantly higher age, ALBI score, and carcinoembryonic antigen (CEA) levels (*P < 0.05*). Both ALBI score (*OR = 0.113, 95% CI: 0.014–0.904*) and age (*OR = 0.935, 95% CI: 0.875–1.000*) were independent predictors of trastuzumab resistance in HER2-positive BC patients. ROC curve analysis showed that the combination of age and ALBI score predicted resistance with an AUC of 0.771 (*95% CI: 0.642–0.900*), sensitivity of 72.7%, and specificity of 77.4%, demonstrating significantly superior predictive performance compared to ALBI score or age alone.

**Conclusion:**

Both ALBI score and age are independent predictors influencing trastuzumab resistance in HER-2 positive BC patients. Their combined use enhances predictive accuracy and may facilitate early identification of patients at increased risk of developing resistance to trastuzumab.

## 1 Introduction

Currently, the global burden of BC continues to rise. As one of the major health threats facing women, this disease has garnered widespread attention due to its high incidence and mortality rates (Bray et al., 2024). Human epidermal growth factor receptor 2 (HER-2) positive BC represents a common molecular subtype, accounting for approximately 15%–20% of all BC cases (Trayes et al., 2021). This subtype exhibits high malignancy, strong invasiveness, poor prognosis, and shorter survival. Trastuzumab is a targeted therapy for HER-2 positive BC. For patients with this subtype, it is commonly used in neoadjuvant therapy and in combination with chemotherapy. Treatment with trastuzumab effectively prolongs survival, reduces the risk of recurrence and metastasis, and significantly improves overall patient prognosis (C et al., 2017) However, in clinical practice, 25%–40% of patients experience tumor progression, recurrence, or metastasis due to trastuzumab resistance during treatment or after standardized therapy, compromising treatment efficacy (Fernández-Nogueira et al., 2020; Ling et al., 2022). Therefore, identifying biomarkers predictive of trastuzumab resistance holds significant importance for the clinical management of HER-2 positive BC.

The Albumin-Bilirubin (ALBI) score is a liver function assessment system that originally developed to predict prognosis in hepatocellular carcinoma patients. Recently, the ALBI score has been applied to predict outcomes in other diseases, such as lung cancer, colorectal cancer, kidney stones, and cardiovascular diseases (Matsukane et al., 2021; Yıldırım et al., 2025; Mo et al., 2025; Qiao et al., 2022). Studies indicate that HER2-positive BC patients face a higher risk of liver metastasis compared to other BC subtypes (Gerratana et al., 2015). Liver metastasis directly compromises liver function, affecting systemic drug metabolism and therapeutic efficacy. Concurrently, liver toxicity induced by trastuzumab is one of its primary adverse effects, primarily manifested as elevated bilirubin levels (Dong et al., 2025). This not only impedes treatment continuity but also damages liver function, The ALBI score provides a simple and direct assessment of liver function. Additionally, aging is often accompanied by physiological characteristics such as impaired immune function, multiple underlying conditions, and reduced drug metabolism capacity, which may also influence resistance to trastuzumab. Previous studies have explored the potential relationship between the ALBI score and primary trastuzumab resistance in HER-2 positive breast cancer patients (Huang et al., 2023). This study will investigate the ALBI score and age predictive value for trastuzumab resistance in HER-2 positive breast cancer patients.

## 2 Information and methods

### 2.1 Study subjects

Patients with HER-2-positive BC using trastuzumab who attended the Department of Thyroid and Breast Surgery of the Affiliated Hospital of Shandong University of Traditional Chinese Medicine from December 2017 to December 2023 were included. Inclusion criteria: 1) Diagnosed with BC by histopathological examination, with pathologic type of HER-2 overexpression (immunohistochemical results showed HER-2 (3+); for HER-2 (2+), further fluorescence *in situ* hybridization (FISH) testing was required to confirm HER-2 gene amplification); 2) Receiving trastuzumab treatment, including preoperative neoadjuvant therapy and postoperative adjuvant chemotherapy; 3) Complete clinical data such as laboratory blood routine and tumor markers. Exclusion criteria: 1) incomplete clinical data; 2) previous history of other malignant tumors and hematologic diseases; 3) combination of primary malignant tumors in other sites.

### 2.2 Research methods

The clinical research big data platform and electronic medical record system of the Affiliated Hospital of Shandong University of Traditional Chinese Medicine were used to obtain relevant information about the patients, including age, laboratory examination results and pathologic diagnosis. Laboratory tests included total bilirubin (TBIL), albumin (ALB), alanine transaminase (ALT), aspartate transaminase (AST), the AST/ALT ratio, these are conventional and reliable liver function markers that are closely associated with drug metabolism, nutritional status, and tumor burden. Carcinoembryonic antigen (CEA), and carbohydrate antigen 125 (CA125), It is a classic tumor marker widely used in breast cancer, commonly employed to assess disease status and prognosis. Pathologic findings included Lymph node metastasis, estrogen receptor (ER) and progesterone receptor (PR) status, and proliferation index (Ki-67) expression, etc. The ALBI score was calculated using the formula: ALBI = (−0.085 × serum ALB level in g/L) + (0.66 × log10 serum TBIL level in μmol/L) (Johnson et al., 2015).

Trastuzumab resistance can be categorized into primary and secondary resistance: Primary resistance refers to disease progression assessed within 3 months of first-line trastuzumab therapy for metastatic BC, or at the first imaging evaluation conducted 8–12 weeks after treatment initiation; alternatively, it denotes the emergence of new lesions within 12 months following adjuvant trastuzumab therapy. Secondary resistance (acquired resistance) refers to disease progression occurring during second-line or subsequent therapy in patients who initially demonstrated disease remission or stabilization on imaging assessment while receiving trastuzumab-containing regimens (W et al., 2011).

This study categorized patients into a resistance group and a non-resistance group based on the development of resistance, collectively designating both primary and secondary resistance as the resistance group.

### 2.3 Statistical methods

Statistical analysis was performed using SPSS25.0(IBM Corp) For normally distributed quantitative data, results are presented as mean ± standard deviation, with intergroup comparisons conducted using t-tests. For non-normally distributed quantitative data, results are presented as median (interquartile range), with intergroup comparisons conducted using the Wilcoxon signed-rank test. Categorical data are presented as counts (percentages), with intergroup comparisons conducted using chi-square tests. All variables with *P < 0.05* in univariate analysis were included in the multivariate logistic regression model to identify independent factors influencing outcomes. For these selected factors, receiver operating characteristic (ROC) curves analysis were plotted to assess diagnostic value, calculating area under the curve (AUC), sensitivity, specificity, and cutoff values. *P < 0.05* was considered statistically significant.

## 3 Results

### 3.1 Incidence of resistance to trastuzumab use in HER-2 positive BC patients

A total of 95 patients with HER-2 positive BC using trastuzumab were included in this study, all of them were female, with an age range of 31–71 years and a mean of (53.15 ± 10.063) years, among which 11 patients (11.6%) developed drug resistance and 84 patients (88.4%) had no drug resistance.

### 3.2 Analysis of factors affecting the development of drug resistance with trastuzumab in HER-2 positive BC patients

Clinical data were compared between HER-2-positive BC patients who developed resistance to trastuzumab and those who did not to screen for factors influencing resistance development. The differences in age, ALBI score and ALB,CEA were statistically significant (all *P < 0.05*), and the differences in other variables were not statistically significant (all *P > 0.05*) (Table 1).

**TABLE 1:** Univariate analysis of HER-2-positive BC patients affecting the development of resistance with trastuzumab.

Variable	Resistant group (n = 11)	Non-resistant group (n = 84)	Statistical value	P-value
Age	58.91 ± 8.871	52.39 ± 10.01	t = 0.824	P = 0.041
ALT	26.82 (16.48∼37.16)	22.29 (18.79∼25.78)	Z = −1.444	P = 0.149
AST	24.56 (18.00∼31.11)	21.09 (18.76∼23.43)	Z = −1.317	P = 0.188
Liver function (AST/ALT)	1.00 (0.77∼1.23)	1.15 (1.03∼1.27)	Z = −0.678	P = 0.498
ALB	37.32 ± 5.21	39.93 ± 3.92	t = −1.997	P = 0.049
TBIL	16.51 (6.88∼26.19)	11.67 (10.75∼12.59)	Z = −0.390	P = 0.697
ALBI	−2.49 ± 0.39	−2.71 ± 0.29	t = 2.158	P = 0.033
CEA	2.85 (2.09∼3.59)	2.51 (2.01∼3.01)	Z = −1.972	P = 0.049
CA125	15.75 (10.69∼20.79)	14.87 (11.85∼17.88)	Z = −1.582	P = 0.114
Tumor location [n (%)]	​	​	X^2^ = 5.430	P = 0.052
Left	9 (81.82)	37 (44.05)	​	​
Right	2 (18.18)	45 (53.57)	​	​
Bilateral	0	2 (2.38)	​	​
Lymph node metastasis [n (%)]	​	​	X^2^ = 0.004	P = 0.951
Metastasis	3 (27.27)	28 (33.33)	​	​
No lymph-node	8 (72.73)	56 (66.67)	​	​
ER [n (%)]	​	​	X^2^ = 0.100	P = 0.752
Positive	6 (54.55)	50 (59.52)	​	​
Negative	5 (45.45)	34 (40.48)	​	​
PR [n (%)]	​	​	X^2^ = 0.135	P = 0.713
Positive	3 (27.27)	32 (38.10)	​	​
Negative	8 (72.73)	52 (61.90)	​	​
Ki-67 [n (%)]	​	​	X^2^ = 0.008	P = 0.928
≥30%	8 (72.73%)	60 (71.43)	​	​
<30%	3 (27.27%)	24 (28.57)	​	​
Recurrence and metastasis status [n (%)]	​	​	X^2^ = 23.981	P = 0.243
Liver metastasis	3 (27.27%)	​	​	​
Metastasis to other sites	4 (36.36%)	​	​	​
Local recurrence	4 (36.36%)	​	​	​

Unconditional binary logistic regression analysis was performed with whether resistance occurred in HER-2 positive breast using trastuzumab cancer patients as the dependent variable, and variables with *P < 0.05* in the univariate analysis as the independent variables. The results of the logistic multivariate analysis showed that age and ALBI were the independent influences on the development of resistance to trastuzumab use in HER-2 positive BC patients (*P < 0.05*) (Table 2).

**TABLE 2:** Multivariate analysis of HER-2-positive BC patients affecting the development of resistance to the use of trastuzumab.

Variable	β	SE	Wald/x^2^	OR	95% CI	P-value
Age	−0.067	0.034	3.899	0.935	0.875–1.000	0.048
ALB	0.152	0.079	3.654	1.164	0.996–1.359	0.059
ALBI	−2.181	1.061	4.225	0.113	0.014–0.904	0.040
CEA	−0.06	0.128	0.222	0.941	0.732–1.211	0.638

## 4 Predictive value of ALBI score in developing resistance to trastuzumab in HER-2 positive BC patients

**FIGURE 1:**
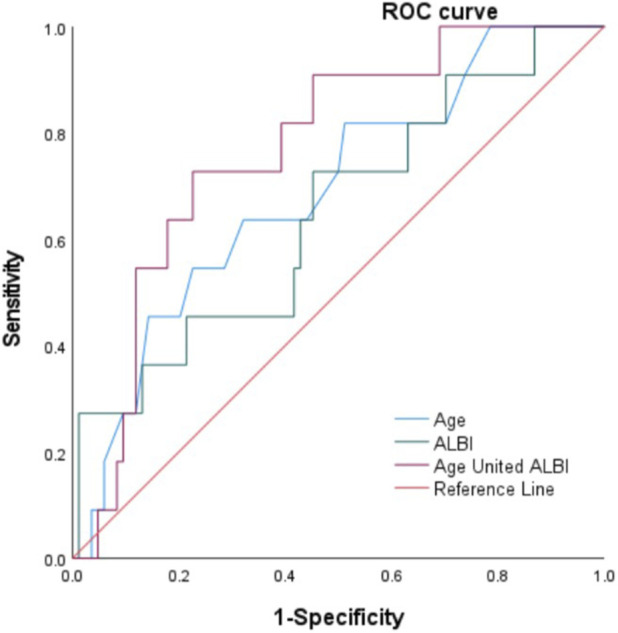
ROC curve of influence of each index on HER-2 positive BC patients using trastuzumab to produce drug resistance.

Plotting the ROC curve analysis showed that the area under the ROC curve (AUC) for age, ALBI score and the combination of the two were 0.690, 0.647 and 0.771, respectively. Assessing the development of resistance to the use of trastuzumab in HER-2-positive BC patients by age, ALBI score and the combination of the two in terms of AUC, sensitivity, specificity, Youden’s index, and 95% CI, respectively. It was suggested that the combination of age and ALBI score had a better diagnostic value for the development of resistance to the use of trastuzumab in HER-2-positive BC patients (Table 3; Figure 1).

**TABLE 3:** Predictive performance of indicators to influence the development of resistance to the use of trastuzumab in patients with HER-2-positive BC.

Variable	AUC	SE	95% CI	P-Value	Sensitivity	Specificity	Youden’s index
Age	0.690	0.082	0.529–0.851	0.041	0.545	0.774	0.319
ALBI	0.647	0.091	0.468–0.826	0.114	0.727	0.548	0.275
Combined (age & ALBI)	0.771	0.066	0.642–0.900	0.004	0.727	0.774	0.501

## 5 Discussion

HER-2-positive BC exhibits biological characteristics such as low differentiation, high invasiveness, and a high rate of distant metastasis. This subtype of BC also presents significant challenges in clinical diagnosis and treatment, including greater therapeutic difficulty and lower long-term survival rates for patients (Boudy et al., 2020). Trastuzumab, as a molecularly targeted therapeutic agent, is a synthetic monoclonal antibody. Its primary mechanism of action involves potent inhibition of HER-2 signaling pathway activity, effectively blocking downstream pathways such as PI3K/AKT/mTOR and mitogen-activated protein kinase/extracellular signal-regulated kinase (MAPK/ERK) pathways downstream of HER-2, thereby inhibiting tumor cell proliferation and promoting apoptosis. Concurrently, it induces antibody-dependent cellular cytotoxicity (ADCC) by recruiting immune cells to eliminate tumor cells and suppressing angiogenesis (Kreutzfeldt et al., 2020), significantly improving the quality of life for HER-2-positive BC patients. However, since HER2-positive BC is inherently an aggressive subtype, trastuzumab can only partially block HER2 signaling. It fails to address issues such as tumor heterogeneity, bypass activation, micro metastasis, and immune evasion. Compounded by drug resistance and overlapping high-risk clinical factors, HER2-positive BC patients treated with trastuzumab still face a high risk of recurrence and metastasis, particularly when trastuzumab serves as the primary therapy (Singer et al., 2008; Koygun et al., 2021). Therefore, early prediction of potential trastuzumab resistance in these patients is crucial for optimizing clinical treatment strategies.

This study revealed that the resistant group exhibited significantly higher mean age and ALBI scores compared to the non-resistant group (P < 0.05). The ALBI score was initially developed to assess liver function and prognosis in hepatocellular carcinoma patients. Recent studies indicate that the ALBI score may also serve as a potential biomarker for predicting prognosis and drug efficacy across multiple malignancies (Huang et al., 2023; Deng et al., 2020). Its calculation formula is straightforward, requiring only two routine laboratory indicators—serum albumin and total bilirubin—to compute the score. Higher scores typically indicate more severe liver function impairment.

Current research indicates that accelerated drug metabolism within the body is one mechanism contributing to drug resistance. As the primary carrier protein in plasma that binds to most drugs, albumin participates in the transport and distribution of drugs throughout the body. When albumin levels decrease, drugs lack the transport and buffering effects provided by albumin, potentially leading to premature metabolic clearance. This hinders the maintenance of effective therapeutic concentrations within the body, thereby compromising treatment efficacy and even inducing drug resistance (Bagheri et al., 2021; Tayyab and Feroz, 2021; Liu and Chen, 2016). As the key organ for albumin synthesis and metabolism, liver dysfunction frequently causes alterations in serum albumin levels, thereby disrupting the pharmacokinetic processes of drugs.

Simultaneously, the liver is also a vital organ for bilirubin metabolism. Elevated serum total bilirubin levels indicate potential disruption to the structure and function of normal hepatocytes, impairing the processes of bilirubin uptake, conjugation, and excretion. Distant tumor metastasis itself is a significant clinical indicator of trastuzumab resistance. Once tumors metastasize to the liver, they can damage liver tissue and disrupt bilirubin metabolism, causing serum total bilirubin levels to deviate from the normal range. Additionally, advancing age in patients is often accompanied by immune system decline and reduced liver and kidney function, which may decrease the body’s ability to metabolize and eliminate drugs, thereby affecting treatment efficacy.

Additional studies indicate that lipid metabolism is closely associated with the development of trastuzumab resistance in HER-2 positive BC. Adipose tissue can influence the sensitivity of HER-2 positive BC cells to targeted therapy by altering the tumor microenvironment through the secretion of inflammatory mediators such as IL-6 (Griner et al., 2013; Duong et al., 2015). Simultaneously, within the tumor microenvironment, adipocytes can transform into cancer-associated fibroblasts (CAFs). These CAFs induce drug resistance in HER2-positive BC cells by secreting multiple cytokines and activating intracellular signaling pathways (Mao et al., 2015; Zhong et al., 2016). Thus, adipose tissue surrounding HER2-positive BC cells not only supplies extracellular lipids but also creates a microenvironment conducive to trastuzumab resistance through inflammatory cytokine secretion and adipocyte trans differentiation. The liver plays a crucial role in lipid metabolism within the body. Disruption of its function may adversely affect lipid metabolism in regulating tumor cell survival, proliferation, invasion, and drug resistance.

The resistant group exhibited significantly higher age, ALBI scores, and CEA levels compared to the non-resistant group, while ALB levels decreased (P < 0.05). However, ALB and CEA levels were not independent predictors of trastuzumab resistance in HER-2-positive BC patients (P > 0.05). This may be because CEA is a non-specific tumor marker. In some patients, its elevation may be influenced by other confounding factors (such as benign gastrointestinal diseases), thereby weakening its independent association with trastuzumab resistance. Albumin levels are significantly influenced by clinical interventions such as diet, intravenous fluid administration, and nutritional support. In some patients, reduced albumin may result from inadequate nutritional intake rather than tumor progression. This non-specific variation precludes albumin from serving as an independent predictor of drug resistance. Furthermore, the relatively small sample size of this study may introduce statistical bias affecting the predictive power of ALB and CEA as independent predictors.

Based on the aforementioned theoretical background, this study evaluated the predictive efficacy of combining the ALBI score with age using ROC curves. The area under the ROC curve for predicting trastuzumab resistance in HER-2-positive BC patients using the combined ALBI score and age was 0.771. Previous studies have demonstrated that high ALBI scores correlate with primary resistance to trastuzumab in HER2-positive BC patients (Huang et al., 2023). This study incorporated data from patients across different regions and healthcare settings, grouping both primary and secondary resistance into a unified resistance cohort. Additionally, participant recruitment criteria, baseline characteristics, and follow-up duration differed from previous reports. Our findings demonstrate that the combined use of ALBI score and age exhibits strong predictive efficacy, thereby providing supplementary evidence to the study by Huang et al. (2023). Huang et al. demonstrated overall clinical applicability at the population level, while our study adds clinically significant age-dependent heterogeneity, further refining and expanding their findings and providing practical new insights for clinical practice.

In summary, the combination of ALBI score and age holds some predictive value for trastuzumab resistance in HER-2 positive BC patients. However, several key questions regarding the mechanisms and influencing factors of trastuzumab resistance remain unresolved, and this study also has limitations. First, systematic analysis of lipid profile parameters was not conducted. As a vital component of cellular metabolism, lipid metabolism is closely associated with trastuzumab resistance. The absence of lipid profile parameters analysis prevents a comprehensive understanding of lipid changes and hinders the identification of potential lipid-related biomarkers or regulatory mechanisms. Future studies incorporating lipid profile parameters analysis are necessary to achieve a more complete understanding. Secondly, as this study is a single-center retrospective analysis with a limited sample size, its conclusions may be subject to certain biases. This study analyzed only selected clinical and pathological factors, failing to comprehensively cover key pathological indicators potentially involved in trastuzumab resistance. Consequently, the sample size and variable coverage exhibit certain limitations. Furthermore, both primary and secondary resistance represent major clinical challenges in trastuzumab therapy. Due to issues such as the small number of patients in the resistance group, this study did not distinguish between primary and secondary resistance. Therefore, future research should conduct multicenter, large-sample prospective studies incorporating more potential predictors to elucidate the mechanisms of trastuzumab resistance, analyze distinctions between different resistance types, and further investigate the predictive value of the ALBI score for these two resistance types. This would provide clinicians with more comprehensive clinical biomarkers, offering robust data support and theoretical basis for more precise and rational application of this drug in practice.

## Data Availability

The original contributions presented in the study are included in the article/supplementary material, further inquiries can be directed to the corresponding author.
